# Deep reinforcement learning for the control of microbial co-cultures in bioreactors

**DOI:** 10.1371/journal.pcbi.1007783

**Published:** 2020-04-10

**Authors:** Neythen J. Treloar, Alex J. H. Fedorec, Brian Ingalls, Chris P. Barnes

**Affiliations:** 1 Department of Cell and Developmental Biology, University College London, London, United Kingdom; 2 Department of Applied Mathematics, University of Waterloo, Waterloo, Canada; 3 UCL Genetics Institute, University College London, London, United Kingdom; Duke University, UNITED STATES

## Abstract

Multi-species microbial communities are widespread in natural ecosystems. When employed for biomanufacturing, engineered synthetic communities have shown increased productivity in comparison with monocultures and allow for the reduction of metabolic load by compartmentalising bioprocesses between multiple sub-populations. Despite these benefits, co-cultures are rarely used in practice because control over the constituent species of an assembled community has proven challenging. Here we demonstrate, *in silico*, the efficacy of an approach from artificial intelligence—reinforcement learning—for the control of co-cultures within continuous bioreactors. We confirm that feedback via a trained reinforcement learning agent can be used to maintain populations at target levels, and that model-free performance with bang-bang control can outperform a traditional proportional integral controller with continuous control, when faced with infrequent sampling. Further, we demonstrate that a satisfactory control policy can be learned in one twenty-four hour experiment by running five bioreactors in parallel. Finally, we show that reinforcement learning can directly optimise the output of a co-culture bioprocess. Overall, reinforcement learning is a promising technique for the control of microbial communities.

## Author summary

In recent years, synthetic biology and industrial bioprocessing have been implementing increasingly complex systems composed of multiple, interacting microbial strains. This has many advantages over single culture systems, including enhanced modularization and the reduction of the metabolic burden imposed on strains. Despite these advantages, the control of multi-species communities (co-cultures) within bioreactors remains extremely challenging and this is the key reason why most industrial processing still uses single cultures. In this work, we apply recently developed methods from artificial intelligence, namely reinforcement learning combined with neural networks, which underlie many of the most recent successes of deep learning, to the control of multiple interacting species in a bioreactor. This approach is model-free—the details of the interacting populations do not need to be known—and is therefore widely applicable. We anticipate that artificial intelligence has a fundamental role to play in optimizing and controlling processes in synthetic biology.

## Introduction

The ability to engineer cells at the genetic level has enabled the research community to make use of biological organisms for many functions, including the production of biofuels [1–3], pharmaceuticals [4] and the processing of waste products [5]. Communities consisting of multiple strains of cells have been shown, in some cases, to be more productive than monocultures at performing processes such as biofuel production [2, 3, 6] and alleviate the problem of metabolic burden that occurs when a large pathway is built within a single cell [7]. For these reasons co-cultures should play a significant role in the advancement of bioprocessing. However, maintaining a co-culture presents its own set of problems. The competitive exclusion principle states that when multiple populations compete for a single limiting resource, a single population with the highest fitness will drive the others to extinction [8]. It has been proven that, under ideal conditions, at most one population can survive indefinitely in a chemostat where multiple cell populations are competing for a single substrate [8]. An additional challenge is that the interactions between different populations of microbes can make long term behaviour in a co-culture difficult to predict [9]; the higher the number of distinct populations, the greater the challenge becomes to ensure system stability [10].

Previously, methods of co-culture population control have been engineered into cells genetically, e.g. using predator-prey systems [11] or mutualism [7, 12]. However, processes such as horizontal gene transfer and mutation make the long term genetic stability of a population hard to guarantee [9], meaning that genetic control methods can become less effective over time. Another potential problem is the increased metabolic load imposed on a cell due to the control genes, which can leave less resources for growth and the production of useful products [13]. These downsides can be avoided by exerting control over the environment, which is the dominant approach in industry. Established techniques are Proportional-Integral-Derivative controllers [14], Model-Predictive-Controllers [15–17] or the development of *ad hoc* feedback laws [18–21]. Here we investigate the viability of reinforcement learning as a complement to these methods.

For our analysis, we use the chemostat model, which provides a standard description of bioprocess conditions. This model is applicable to a wide range of other systems where cell or microorganism growth is important, including wastewater treatment [22] and the gut microbiome [23]. Such systems can be especially difficult to control because they are often equipped with minimal online sensors [24], limiting the effectiveness of classical control techniques that are hampered by infrequent or delayed system measurements [20, 25].

**Fig 1:**
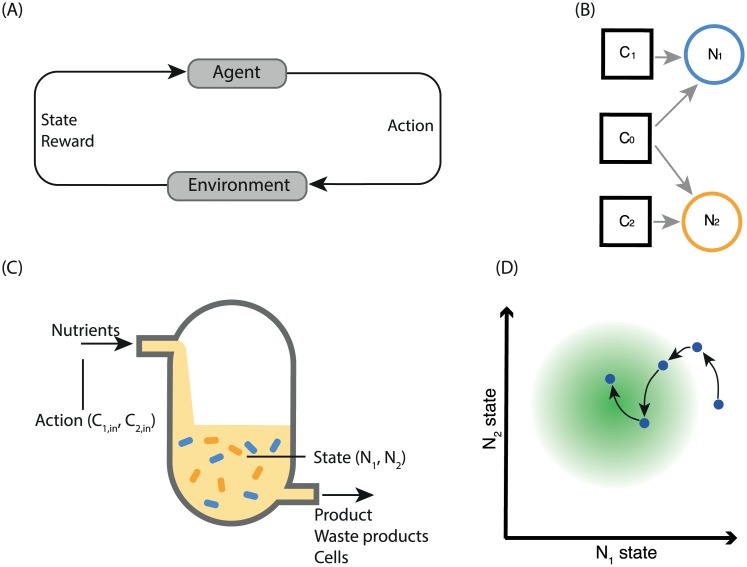
Reinforcement learning for the control of two auxotrophic species in a chemostat. (A) The basic reinforcement learning loop; the agent interacts with its environment through actions and observes the state of the environment along with a reward. The agent acts to maximise the total reward it receives (the return). (B) System of two auxotrophs dependent on two different nutrients, with competition over a common carbon source. (C) Diagram of a chemostat. The state observed by the reinforcement learning agent is composed of the populations of two strains of bacteria; the actions taken by the agent control the concentration of auxotrophic nutrients flowing into the reactor. (D) Representative system trajectory. The agent’s actions, taken at discrete time-points (circles), influence the state dynamics (black arrows), with the aim of fulfilling the reward condition (moving to the centre of the green circle). The state is comprised of the (continuously-defined) abundance of two microbial populations, **N**_1_ and **N**_2_. The agent’s actions dictate the rate at which auxotrophic nutrients flow into the reactor. At each time-step, the agent’s reward is dependent on the distance between the current state and the target state.

Reinforcement learning is a branch of machine learning concerned with optimising an agent’s behaviour within an environment. The agent learns an optimal behaviour policy by observing environmental states and selecting from a set of actions that change the environment’s state (Fig 1A). The agent learns to maximise an external reward that is dependent on the state of the environment. The training of a reinforcement learning agent is often broken up into *episodes*. An episode is defined as a temporal sequence of states, rewards and corresponding actions (generated by the agent interacting with the environment) until a terminal state is reached. The total reward obtained during an episode is called the return. For this study, we used a data-efficient variant of reinforcement learning called Neural Fitted Q-learning [26–28] (see Methods).

Much reinforcement learning research has been done on video games [29] due to the availability of plentiful training data. However, it is also seeing application to more practical problems in the sciences, including the optimisation of chemical reactions [30] and in deriving optimal treatment stategies for HIV [31] and sepsis [32]. A partially supervised reinforcement learning algorithm has also been applied to a model of a fed-batch bioreactor containing a yeast monoculture [33].

Here we develop a control scenario in which the growth of two microbial species in a chemostat is regulated through the addition of nutrients *C*_1_ and *C*_2_ for which each species is independently auxotrophic (Fig 1B and 1C). The influx of each nutrient is controlled in a simple, on-off manner (bang-bang control). At each time point, the agent decides, for each auxotrophic nutrient, whether to supply this nutrient to the environment at the fixed inflow rate over the subsequent inter-sample interval. This constitutes the set of possible actions. A constant amount of carbon source, *C*_0_, is supplied to the co-culture. We define the system state as the population levels of each population in the chemostat (assumed to be measured using fluorescence techniques). The objective is either to maintain specific population levels or to maximize product output. A corresponding reward is given that depends on the distance of the population levels from the target value or as a function of product output. The populations evolve continuously, and the reward is likewise a continuous function of the state. In contrast, the agent’s actions are discrete (bang-bang), and are implemented in a sample-and-hold strategy over a set of discrete sampling times. A visual representation of a two-population case is shown in Fig 1D.

Below, we illustrate that an agent can successfully learn to control the bioreactor system in the customary episodic manner and is robust to differing initial conditions and target set-points. Secondly, we compare our reinforcement learning approach to proportional integral control, both working in a model free way on simulated data, and show that the learning approach performs better in situations where sampling is infrequent. We then show that the agent can learn a good policy in a feasible twenty four hour experiment. Finally, we demonstrate that reinforcement learning can be used to optimise productivity from direct observations of the microbial community. Traditional proportional integral control could only be applied to such a case via a model of the system, or with additional measurement data from further online sensors.

## Results

### Reinforcement learning can be used to control the bioreactor system

We developed a parameterised model to simulate the growth of two distinct *E. coli* strains in a continuous bioreactor, with glucose as the shared carbon source, *C*_0_, and arginine and tryptophan as the auxotrophic nutrients *C*_1_ and *C*_2_ (Fig 1B and 1C, Methods, Table 1). Episodic Fitted Q-learning (Algorithm 2, Methods) was then applied to the model of the system. The reward was selected to penalize deviation from target populations of [*N*_1_, *N*_2_] = [20, 30] × 10^9^ cells L^−1^. Specifically, the reward function was: $r=\frac{1}{10}(1-\frac{1}{2}\left(\frac{\left|N_{1}-N_{1}^{target}\right|}{20\times 10^{9}}+\frac{\left|N_{2}-N_{2}^{target}\right|}{30\times 10^{9}}\right))$. The scaling of $\frac{1}{10}$ was selected to ensure a maximum possible reward of 0.1, which helped prevent network instability. (Negative rewards below -0.1 are possible; however due to the system dynamics they rarely occurred and did not effect training performance). The contribution of each population was scaled according to its target value so that each contributed proportionally to the reward. This prevented the contribution to the reward function from one strain becoming insignificant if its target value was considerably smaller than the other. The absolute error was chosen because it is continuous and differentiable (except when populations are at the target value) and has a unique minimum, all properties that are favourable for reinforcement learning in continuous state spaces. Absolute error was chosen over the squared error so that the reward gradient didn’t diminish in the region near the target. The reward function is based on target population levels because we have already assumed that these are measurable through, for example, fluorescence measurement. Selection of a target set-point in state space is also an approach widely used with more traditional control techniques and so allows for direct comparison to these.

**Table 1:** Double auxotroph system. Parameter values used for simulations of a system consisting of two auxotrophic populations of bacteria with competition for nutrients. *μ*_*max*_ values were chosen using values from the literature [60] as a guide.

Parameter	Description	Value	Unit	Source
**C**_0,*in*_	Reservoir concentration of carbon source	1	g L^−1^	Experimentally controllable
q	Flow rate	0.5	h^−1^	Experimentally controllable
*γ*_0_	Yield coefficient for common substrate	4.8 × 10^11^	cells g^−1^	[58]
*γ*_1_	Yield coefficient for arginine	5.2 × 10^11^	cells g^−1^	[59]
*γ*_2_	Yield coefficient for tryptophan	4.4 × 10^11^	cells g^−1^	[59]
***μ***_*max*,1_	Maximum growth rate	1	h^−1^	[60]
***μ***_*max*,2_	Maximum growth rate	1.1	h^−1^	[60]
**K**_*s*,0_	Saturation constant for the carbon source	6.85 × 10^−5^	g L^−1^	[59]
**K**_*s*,1_	Saturation constant for arginine	4.9 × 10^−4^	g L^−1^	[59]
**K**_*s*,2_	Saturation constant for tryptophan	1.02 × 10^−7^	g L^−1^	[59]

**Fig 2:**
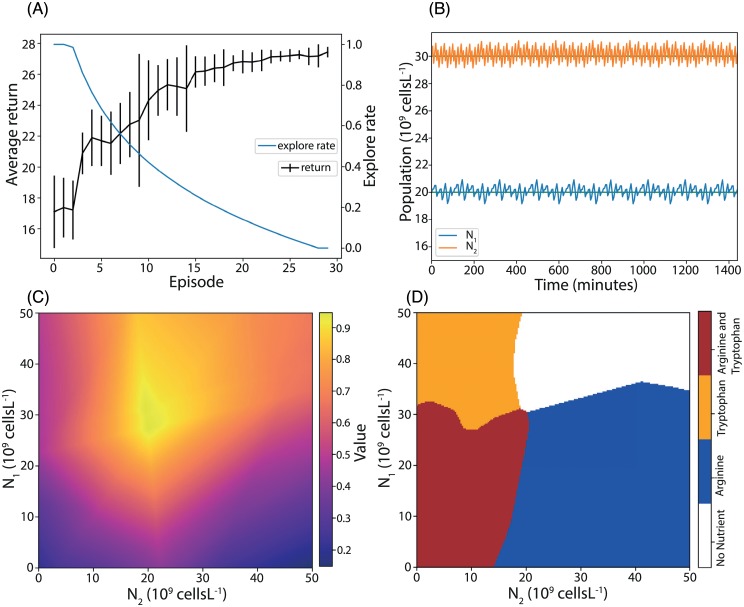
Reinforcement learning applied to the bioreactor system. (A) Performance of the agent improves and the explore rate decreases during training. The average of the return over twenty training replicates is plotted; error bars represent one standard deviation. (B) System behaviour under control of a trained agent for a twenty four hour period. Populations are maintained near target values (green lines). (C) Heatmap of the learned state value function; values are maximal at the target. (D) A learned state action plot, showing the agent’s learned action (coloured regions) over the state space.

The agent was trained on thirty sequential episodes, this provided enough data for the agent to learn while not being prohibitive in terms of computational time. Each episode was twenty four hours long with sampling and subsequent action choice every five minutes. The initial system variables of the chemostat for each episode were [*N*_1_, *N*_2_] = [20, 30] × 10^9^ cells L^−1^ and [*C*_0_, *C*_1_, *C*_2_] = [1, 0, 0] g L^−1^. The explore rate was initially set to *ϵ* = 1 and decayed as *ϵ* = 1 − log_10_(*aE*) where *E* is the episode number, starting at 0, and *a* = 0.3 is a constant that dictates the rate of decay. A minimum explore rate of *ϵ* = 0 was set and was reached by the end of training. Fig 2A shows the training performance of twenty replicate agents, each trained over thirty episodes. The twenty agents converged to a mean final return of 27.4 with a standard deviation of 0.33. The theoretical maximum return is 28.8; all twenty agents were thus able to learn near optimal policies despite being restricted to bang-bang control. The population curve in Fig 2B shows the system behaviour when under control of a representative agent trained in one of the replicates (for all twenty replicates see S1 Fig). The population levels track the targets, with some jitter as expected with a bang-bang controller. Fig 2C shows the value function learned by this representative agent at the end of training, indicating its assessment of the total return from each point in state space. As expected, the value peaks at the target point. The corresponding state-action plot, Fig 2D, shows that the agent has adopted a simple, intuitive feedback law: add the specific nutrient needed by a strain when its population level is below the target and refrain from adding the nutrient if it is above the target. From these results, we conclude that reinforcement learning can be successfully applied to the chemostat system with a practical inter-sampling period of five minutes, as predicted (see Methods).

### Reinforcement learning is robust to different initial conditions and targets

To verify that our algorithm is robust to different initial conditions and different target populations we began by choosing a range of initial population values: [5, 10, 40, 50] × 10^9^ cells L^−1^ and two different targets: [20, 30] × 10^9^ cells L^−1^ and [30, 20] × 10^9^ cells L^−1^. For every combination of initial populations and target, a Fitted Q-agent was trained in the same manner as in the previous section. This was repeated three times. One of the three population curves from each experiment is shown in S2 Fig, the corresponding actions for the first 600 minutes of the simulation are shown in S3 Fig and the average return across each of the three repeats is shown in S4 Fig. On 2 of the 96 total replicate runs the agent failed to maintain the populations at the target levels. This happened with the target: [30, 20] × 10^9^ cells L^−1^ with initial conditions [*N*_1_, *N*_2_] = [5, 5] × 10^9^ cells L^−1^ and [*N*_1_, *N*_2_] = [5, 10] × 10^9^ cells L^−1^. Intuitively these represent two of the most challenging combinations, where the target has the slower growing strain (*N*_1_) above the faster growing strain (*N*_2_) and in which both populations are at low initial values.

### Reinforcement learning outperforms proportional integral control for long sampling periods

**Fig 3:**
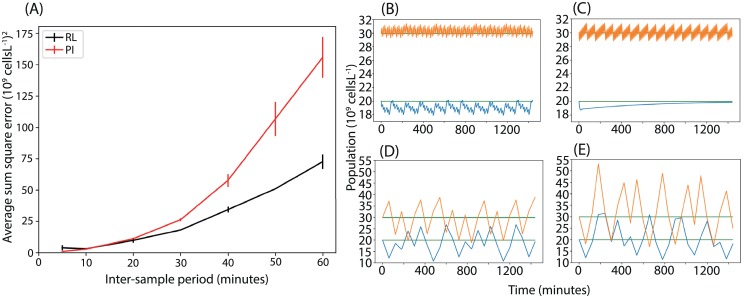
Comparison of reinforcement learning and proportional integral controllers. (A) The scaled average sum square error between the system state and the target. For long inter-sample periods, the reinforcement learning controller outperforms the proportional integral controller. The sum square error was calculated from population values that were scaled by a factor of 10^−9^. (B-C) Population time-courses under the reinforcement learning and proportional integral controllers respectively, with a five minute inter-sampling time. (D-E) Population time-courses under the reinforcement learning and proportional integral controllers respectively, with a sixty minute inter-sampling time. Here the proportional integral controller allows the populations to stray further from their target values.

As a comparison to a standard control approach, the reinforcement learning controller was compared to a traditional proportional integral controller. The controllers differ in that the proportional integral controller implements feedback over a continuous action space, whereas the reinforcement learning controller uses bang-bang control. For both controllers thirty episodes of data were generated, each twenty-four hours long, for a range of sampling-and-hold intervals: *t*_*s*_ = [5, 10, 20, 30, 40, 50, 60] mins by starting with initial system variables [*N*_1_, *N*_2_] = [20, 30] × 10^9^ cells L^−1^ and [*C*_0_, *C*_1_, *C*_2_] = [1, 0, 0] g L^−1^ and sampling random input concentrations *C*_1_, *C*_2_ from [0, 0.1] g L^−1^. For each choice of sampling frequency, the reinforcement learning agent was trained using Fitted Q-iteration (Algorithm 1, Methods) on the dataset of thirty randomly generated episodes, while the proportional integral controller was tuned on an input-output model of the system derived from the same dataset (see Methods). The performance of the two controllers is illustrated in Fig 3, which shows how the performance depends on the choice of sampling frequency. For inter-sampling intervals longer than five minutes, the reinforcement learning controller outperforms the proportional integral controller. We conclude that reinforcement learning can produce comparable and even better performance, with the potential added advantage of a simpler implementation (the proportional integral controller employs continuous actions, whereas the reinforcement learning controller uses only bang-bang control). Moreover, for microbial chemostat systems that are difficult or expensive to sample at high frequency, reinforcement learning could be the preferred option.

### A good policy can be learned online using parallel bioreactors

**Fig 4:**
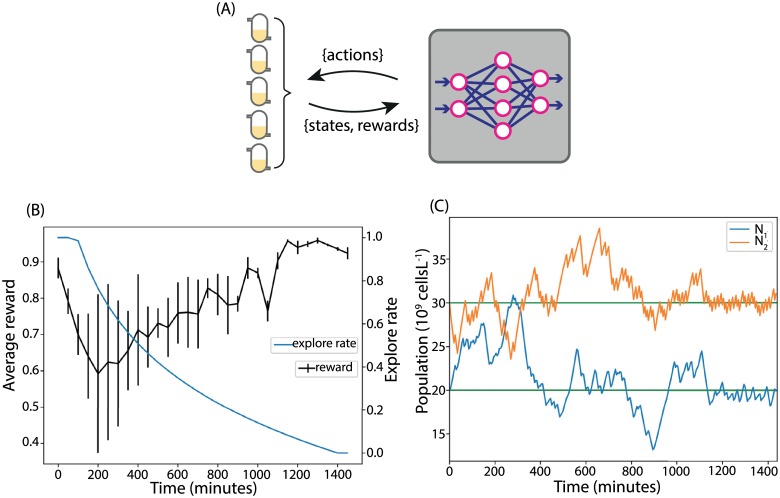
Learning a policy in twenty four hours. (A) A reinforcement learning agent was trained online on a model of five parallel chemostats for twenty four hours. (B) The average reward received from the environments. By the end of the simulation all five chemostats were moved to the target population levels with very litte standard deviation in reward. (C) The population curve from one of the chemostats. During the exploration phase the population levels vary and random actions are taken, as the explore rate decreases they move to the target values.

A barrier to the use of reinforcement learning in real world applications is the amount of data required. Experimental systems do exist that would allow one to gather the necessary data to train an agent in the manner demonstrated above [41]. However, we aim to lower the barrier of entry so that our method can be implemented in low cost bioreactors. For example, the development of a low cost turbidostat capable of running eight cell culture experiments in parallel, demonstrated on time periods up to 40 hours [42] presents a realistic scenario for a cell biology lab. We next show that Online Fitted Q-learning, a variant of Fitted Q-learning adapted to run in an online manner (Algorithm 3, Methods), can learn to control the chemostat system using an amount of data realistically obtainable in a single experiment. We trained an agent online on five chemostat models running in parallel. Each modelled the system described in Fig 1B and 1C and was run for the equivalent of twenty-four hours of real time. The agent took an action every five minutes, making an independent decision for each of the five chemostats from a single policy learned from experience gathered from all models. The reward was observed and the value function updated by the agent every ten time steps, using all experience gathered up to that time (Fig 4A). As in the previous sections, the initial microbial populations were set to the target value of [*N*_1_, *N*_2_] = [20, 30] × 10^9^ cells L^−1^ and the initial concentrations of the nutrients were [*C*_0_, *C*_1_, *C*_2_] = [1, 0, 0] g L^−1^. Fig 4B shows the online reward the agent received from the five chemostats. The initial reward was high, due to the initial populations being set to the target values. As the agent explored, the reward decreased and the standard deviation between the parallel chemostats increased because the agent took independent exploratory actions in each chemostat and drove them into different regions of state space. As time progressed, the reward from all five chemostats increased and the standard deviation decreased because the agent learned and moved all populations closer to the target. A pair of representative population time-courses is shown in Fig 4C (all five are shown in S5 Fig). From these results, we conclude that Online Fitted Q-learning can be used to learn a policy in a data-efficient, online manner.

### The yield of a community-based product can be directly optimised

**Fig 5:**
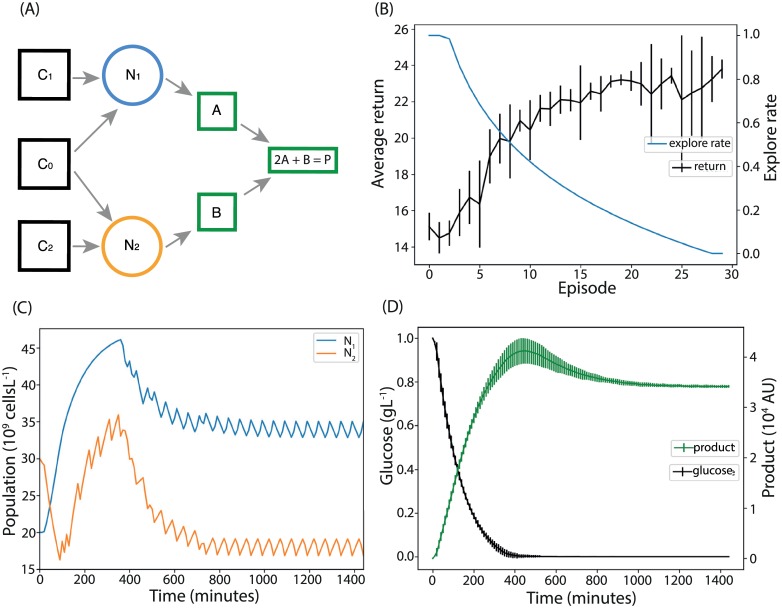
Using reinforcement learning to optimise product output. (A) Each microbial population produces an intermediate; these react to produce the desired product. (B) Training performance of ten reinforcement learning agents trained to optimise product output. (C) The resulting population curves of the system under control of a representative agent. The populations reach and then are maintained at the optimal level for product production. (D) The levels of carbon and product inside the chemostat. After the initial phase all carbon is being consumed. The levels of product peak as all initial carbon is used, then reach a level supported by the carbon supply to the reactor.

To demonstrate the ability of reinforcement learning to directly optimise the output of a community-based bioprocess, the system in Fig 5A was modelled. Here, each microbial strain produces an intermediate product; *N*_1_ produces *A* and *N*_2_ produces *B*, each at a rate of 1 molecule per cell per hour. Factors *A* and *B* react to a product *P*, via the reaction 2*A* + *B* → *P*, which is presumed rapid. Consequently, the optimal state of the system has population ratio *N*_1_: *N*_2_ = 2: 1, with the populations at the maximum levels that the chemostat can support, which in our model means that all the carbon source, *C*_0_, is being consumed. In this case, we set the agent’s reward to be proportional to the instantaneous production rate of the bioreactor. We again take the observed state and the available actions to be the population levels and the bang-bang auxotrophic nutrient inflow rates, respectively. We set the initial populations to [*N*_1_, *N*_2_] = [20, 30] × 10^9^ cells L^−1^ and initial nutrient concentrations to [*C*_0_, *C*_1_, *C*_2_] = [1, 0, 0] g L^−1^ as before. The initial levels of *A*, *B* and *P* were all 0. Ten replicate agents were trained using Episodic Fitted Q-learning (Algorithm 2, Methods). The sample-and-hold interval was increased to ten minutes, which improved learning performance by giving sufficient time for the agent’s actions to affect production rate. Performance in terms of the return is shown in Fig 5B. The average ratio of the population levels in steady state (the last 440 minutes of the simulation), over all agents, was 1.99 (with s.d. 0.08), showing convergence to near optimal populations in all replicates. A representative population time-course is shown in Fig 5C (for all time-courses see S6 Fig). Likewise, the average final concentration of the carbon source was 0.11% (s.d. 0.015%) of the source concentration, showing that in all cases the total population was close to the carrying capacity of the chemostat. As shown in Fig 5D, the replicates showed very little deviation in the final product output rate. However, in the initial phase of moving and stabilising the populations to the optimal levels, there is significant deviation. This suggests that most of the deviation in return shown in Fig 5B is due to this initial stabilising phase and not to the final phase the agents reached. From this analysis, we conclude that the reinforcement learning agent can learn to move the system to,—and keep it at—the near optimal state for product formation in a model free way.

## Discussion

We have applied deep reinforcement learning, specifically Neural Fitted Q-learning, to the control of a model of a microbial co-culture, thus demonstrating its efficacy as a model-free control method that has the potential to complement existing techniques. We have shown that reinforcement learning can perform better than the industry standard, PI control, when faced with long sample-and-hold intervals. In addition, we showed that the data efficiency of Neural Fitted Q-learning can be used to learn a control policy in a practically feasible, twenty-four hour experiment. Reinforcement learning is most often used in environments where data is cheap and effectively infinitely available. Importantly, our results shows that reinforcement learning can also be realistically used to control microbial co-cultures in low cost bioreactors. Finally it is shown that the output of a bacterial community can be optimised in a model free way using only knowledge of microbial population levels and the rate of product output, showing that industrial bioprocess optimisation is a natural application of this technique.

In this work we have developed the approach based upon a chemostat model of a bioreactor. The same approach could be applied in a range of other culture environments. Over the past several years, a number of low-cost bioreactors have been developed that can operate as both turbidostats or as chemostats [41–44]. One such system has 78 chambers running in parallel; easily producing the high volume of data required to train our agent [41]. Another system incorporates online measurement of multiple fluorescence channels, facilitating state measurements at faster intervals than human sampling would allow [44]. Similar devices have been made at a smaller scale, using microfluidics capable of running batch, chemostat and turbidostat cell cultures [45, 46]. These have been applied to high-throughput gene analysis [45, 46], elucidating the relationship between population density and antibiotic effectiveness [47]. The development of a morbidostat facilitated the investigation into the evolution of resistance to antibiotics [48]. As these devices become more widely available, intelligent control methods could be used to explore these important topics while enabling additional layers of complexity, such as multiple competing species or environmental variation.

Here we adopted auxotrophy as our mechanism for control. The utility of this approach has been highlighted by previous studies of microbial communities [49–52]. Other methods of controlling strain growth or competitiveness could also be used as long as they can be externally controlled by the agent e.g. independent carbon sources [53], induced lysis [54] or growth arrest [55].

It should be noted that any attempt to control microbial populations may give rise to mutations. Because reinforcement learning approaches involve continual updates of the agent’s policy, our method has the capacity to adapt to evolutionary changes in the growth dynamics. Understanding how to control populations of evolving species is crucial for preventing the development of antibiotic resistance [56] and the design of chemotherapy regimens [57]. Dynamic programming, the model-based analogue of reinforcement learning, has been used to solve the optimal control problem in both of these scenarios.

Overall, we have demonstrated the potential for control of multi-species communities using deep reinforcement learning. As synthetic biology and industrial biotechnology continue to adopt more complex processes for the generation of products from fine chemicals to biofuels, engineering of communities will become increasingly important. This work suggests that leveraging new developments in artificial intelligence may be highly suited to the control of these valuable and complex systems.

## Methods

### A mathematical model of interacting bacterial populations in a chemostat

We develop a general model of *m* auxotrophs growing and competing in a chemostat. The model captures the dynamics of the abundance of each species (*m*-vector **N**), the concentration of each auxotrophic nutrient (*m*-vector **C**), and the concentration of the shared carbon source (scalar *C*_0_). A sketch of the two-species case is shown in Fig 1B and 1C.

The rate of change of the concentration of the shared carbon source is given by:

$$\begin{array}{c} \frac{d}{dt}C_{0}\left(t\right)=q\left(C_{0,in}-C_{0}\left(t\right)\right)-\sum_{i=1}^{m}\frac{1}{\gamma_{0,i}}\mu_{i}\left(t\right)N_{i}\left(t\right) \end{array}$$ (1)

where *γ*_0_ is a vector of the bacterial yield coefficients for each species, *C*_0,*in*_ is the concentration of the carbon source flowing into the bioreactor, *μ* is the vector of the growth rates for each species, and *q* is the flow rate. The parameters are found in Table 1.

The concentration of each auxotrophic nutrient **C**_*i*_ is given by:

$$\begin{array}{c} \frac{d}{dt}C_{i}\left(t\right)=q\left(C_{i,in}\left(t\right)-C_{i}\left(t\right)\right)-\frac{1}{\gamma_{i}}\mu_{i}\left(t\right)N_{i}\left(t\right) \end{array}$$ (2)

where *γ* is a vector of bacterial yield for each auxotrophic species with respect to their nutrient and **C**_*in*_ is a vector of the concentration of each nutrient flowing into the reactor (which is the quantity controlled by the reinforcement learning agent). Note that we assume all the auxotrophs are independent, i.e. each auxotrophic nutrient is only used by one population.

The growth rate of each population is modelled using the Monod equation:

$$\begin{array}{c} \mu_{i}=\mu_{max,i}\frac{C_{i}}{K_{s,i}+C_{i}}\frac{C_{0}}{K_{s0,i}+C_{0}} \end{array}$$ (3)

where ***μ***_*max*_ is a vector of the maximum growth rate for each species, **K**_*s*_ is a vector of half-maximal auxotrophic nutrient concentrations and **K**_*s*0_ is a vector of half-maximal concentrations *C*_0_ for the shared carbon source. Finally, the growth rate for each population is determined as:

$$\begin{array}{c} \frac{d}{dt}N_{i}\left(t\right)=\left(\mu_{i}\left(t\right)-q\right)N_{i}\left(t\right). \end{array}$$ (4)

### Neural Fitted Q-learning algorithm

A value function is learned which maps state action pairs to values. Here a state transition is defined as the tuple (*s*_*t*_, *a*_*t*_, *r*_*t*_, *s*_*t*+1_) specifying, respectively, the system state, action taken, reward received at time *t*, and the state of the system at time *t* + 1. From a sequence of these state transitions a sequence of Q-learning targets is created according to:

$$\begin{array}{c} Q{\left(s_{t},a_{t}\right)}_{target}=r_{t}+\gamma \;\underset{a}{\text{max}}\;Q\left(s_{t+1},a\right) \end{array}$$ (5)

Here, the term max_*a*_ *Q*(*s*_*t*+1_, *a*), where *a* is an action that can be taken by the agent, gives an estimate of the total future reward obtained by entering state *s*_*t*+1_. This is weighted by γ, the discount factor, which dictates how heavily the possible future rewards weigh in on decisions. In this work, the discount factor was set to 0.9, which is a common first choice in reinforcement learning applications. A neural network is trained on the set of inputs {(*s*_*t*_, *a*_*t*_)∀*t*} and targets {*Q*(*s*_*t*_, *a*_*t*_)_*target*_∀*t*} generated from all training data seen so far (Algorithm 1). In Episodic Fitted Q-learning this was done after each episode (Algorithm 2) while in Online Fitted Q-learning this was done after each update interval (Algorithm 3).

**Algorithm 1** Fitted Q-iteration

1: input: {(*s*_*t*_, *a*_*t*_, *r*_*t*_, *s*_*t*+1_) ∀*t*}

2: iter = 0

3: N = 10            ⊳ number of Fitted Q-iterations

4: **while** iter < N **do**

5:  reinitialise Q network

6:  *inputs* = {*s*_*t*_ ∀*t*}

7:  *targets* = {*r*_*t*_ + *γ* max_*a*_ *Q*_*iter*_(*s*_*t*+1_) ∀*t*}

8:  train Q network on (*inputs*, *targets*)→*Q*_*iter*+1_

9:  iter = iter + 1

10: **end while**

11: **return** *Q*_*N*_

**Algorithm 2** Episodic Fitted Q-learning

1: iter = 0

2: N = 30                     ⊳ number of episodes

3: tmax = 288             ⊳ number of timesteps in each episode

4: **while** iter < N **do**

5:  **for** i in 1 to tmax **do**

6:   *a* = *π*(*s*_*t*,*env*_, *Q*_*N*_)          ⊳ get action based on current policy

7:   (*s*_*t*_, *a*_*t*_, *r*_*t*_, *s*_*t*+1_) = *env*.*step*(*a*)   ⊳ interact with env and observe transition

8:   *M* ← *M* + (*s*_*t*_, *a*_*t*_, *r*_*t*_, *s*_*t*+1_)          ⊳ add transition to memory

9:  **end for**

10:  *Q*_*N*_ = Fitted_Q_iteration(*M*)           ⊳ update agent’s policy

11: **end while**

12: **return** *Q*_*N*_

**Algorithm 3** Online Fitted Q-learning

1: input: *envs*           ⊳ set of environments to learn from

2: N = 288                ⊳ 24hrs of 5min time steps

3: update_frequency = 10     ⊳ update the policy every 10 time steps

4: iter = 0

5: **while** iter < N **do**

6:  **for** each env **do**

7:   *a* = *π*(*s*_*t*,*env*_, *Q*_*N*_)        ⊳ get action based on current policy

8:   (*s*_*t*_, *a*_*t*_, *r*_*t*_, *s*_*t*+1_) = *env*.*step*(*a*) ⊳ interact with env and observe transition

9:   *M* ← *M* + (*s*_*t*_, *a*_*t*_, *r*_*t*_, *s*_*t*+1_)        ⊳ add transition to memory

10:  **end for**

11:  **if** iter % update_frequency ⩵ 0 **then**

12:   *Q*_*N*_ = Fitted_Q_iteration(*M*)       ⊳ update agent’s policy

13:  **end if**

14:  iter = iter + 1

15: **end while**

16: **return** *Q*_*N*_

We use an *ϵ*-greedy policy in which a random action is chosen with probability *ϵ* and the action *a* = max_*a*_ *Q*(*s*_*t*_, *a*) is chosen with probability 1 − *ϵ*. The explore rate *ϵ* was set to decay exponentially as training progressed. *ϵ*-greedy is a widely used policy that has been proven effective [29, 34] and is easy to implement.

The state variables considered by the algorithm were the continuous populations of each species of microbe. The agent acted as a bang-bang controller with respect to each input nutrient, giving 2^n^ possible actions, where *n* is the number of nutrients. (In this work, *n* = 2).

The neural network that was used to estimate the value function consisted of two hidden layers of 20 nodes, following the approach in previous work [27]. Each node in the hidden layers used the ReLU activation function. The input layer had *n* nodes, one for each microbial strain; the linear output layer had 2^*n*^ nodes, one for each available action. We used the Adam optimiser [35], because of its ability to dynamically adapt the learning rate, which is favourable when implementing reinforcement learning with a neural network [36]. The populations levels were scaled by a factor of 10^−5^ before being entered into the neural network; this generated values between 0 and 1 (with units 10^6^ cells L^−1^) and prevented network instability.

Python version 3.6.7 was used for all reinforcement learning code, available at http://www.python.org. The odeint function of SciPy (version 1.3.1) [37] was used to numerically solve all differential equations. The neural network was implemented in Google’s TensorFlow (version 1.13.1) [38]. Numpy (version 1.16.14) was used throughout [39]. The code and examples are available on GitHub: https://github.com/ucl-cssb/ROCC.

### Reinforcement learning parameter tuning

We carried out preliminary investigations to calibrate parameters for the reinforcement learning controller, as follows.

#### Minimum inter-sampling period

The theoretical convergence guarantees of reinforcement learning assume that it is applied to a Markov decision process [61]. The two-strain chemostat system we use here has five state variables: two auxotrophic nutrient concentrations, the concentration of carbon source and the two microbial population levels. Only the population levels are known to the agent, meaning the system is only partially observed and is hence not a Markov decision process. There are methods to extend reinforcement learning to partially observed Markov decision processes, including incorporating time series information using a recurrent network [34], keeping track of approximate belief states of the hidden variables [62] or using Monte-Carlo methods [63]. To assess whether these computationally expensive methods would be required, we determined the minimum sample-and-hold interval that allowed the agent to accurately predict the reward resulting from a chosen action. (Intuitively, this can be thought of as the minimum sample-and-hold interval in which an action has time to have an effect on the observed states (the population levels).) To determine this minimum interval length, we first generated system trajectories of (*s*_*t*_, *a*_*t*_, *r*_*t*_) resulting from random actions. The agent was trained on these sequences to predict the reward *r*_*t*_ from the state action pairs (*s*_*t*_, *a*_*t*_). We repeated this process one hundred times for each of the following sampling times: [1, 2, 3, 4, 5, 10] minutes. The results, shown in S7A Fig, indicate that at time steps lower than four minutes the agents are unable to accurately predict the reward received from the state and action, meaning reinforcement learning cannot be effective. However at four minutes and above the reward prediction is accurate. We concluded that by using intervals of four minutes or longer, the sophisticated non-Markovian methods mentioned above would not be required for this application. S7B Fig shows the reward prediction for both one- and five-minute time steps, showing that the agent performs well for five minutes intervals and poorly for one minute intervals.

#### Number of Fitted Q-iterations to avoid over fitting

To determine how many Fitted Q-iterations to implement, we generated sequences of (*s*_*t*_, *a*_*t*_, *r*_*t*_) of varying lengths by interacting with the chemostat system using randomly chosen actions. Fitted Q-agents were trained to predict the instantaneous reward *r*_*t*_ (by setting γ = 0) from the state-action pair (*s*_*t*_, *a*_*t*_). This was done with the rationale that the ability to correctly predict the instantaneous reward from a state-action pair is a requirement for the ability to predict the value from a state-action pair. We determined the training and testing error for each Fitted Q-iteration, with a maximum of 40 iterations. S8 Fig shows the results of repeating this process 100 times for each sequence length. The data reveal clear overfitting for the datasets shorter than 200 time steps long and a reduction in testing error as the sequence length increases (i.e. with more training data). For each sequence length, the training process with 4 fitted Q iterations gave the smallest testing error (except for 100 training timesteps, where 5 iterations performed marginally better). With a training set of 200 time steps, no significant overfitting occurred.

#### Number of Fitted Q-iterations for value convergence

Another consideration is how many Fitted Q-iterations are required for the values to converge via bootstrapping. For this analysis, we generated 100 sequences of (*s*_*t*_, *a*_*t*_, *r*_*t*_), each one thousand time steps long, in the same manner as the previous section. For each sequence, the actual values were calculated and Fitted Q-iteration was used to obtain predicted values. After each Fitted Q-iteration, the error between the predicted and actual values was recorded. As shown in S9 Fig, the values converge after about ten iterations. Using this and the information from the previous section, the number of Fitted Q-iterations was chosen depending on the length of the agent’s memory to both prevent overfitting and to allow convergence via bootstrapping. For all episodic Q-Learning the number of Fitted Q-iterations was set to 10 as the agent’s memory always contains at least one episode of 288 transitions. For online Q-Learning the number of Fitted Q-iterations was set to 4 if there were less than 100 transitions in the agent’s memory, 5 if there were 100-199 transitions and 10 if there were 200 or more transitions.

### Proportional integral controller tuning

For the comparison between reinforcement learning and PI control, we tested a range of sample-and-hold intervals ([5, 10, 20, 30, 40, 50, 60] mins). For each choice of sampling interval, we generated thirty, twenty-four hour long episodes, each starting with initial system variables [*N*_1_, *N*_2_] = [20, 30] × 10^9^ cells L^−1^ and [*C*_0_, *C*_1_, *C*_2_] = [1, 0, 0] *g* *L*^−1^ and selecting actions randomly from [0, 0.1] *g* *L*^−1^. These thirty episodes were used as training data for the Fitted Q-agent. From each dataset, an input-output model was constructed using the plant identification function in the PID tuner app of MATLAB’s Simulink toolbox, which allows the identification of an input-output model for any input-output dataset. Here, the randomly chosen actions were used as input and the resulting populations (scaled by a factor of 10^−10^) were taken as output. The model was a state space model, of order chosen by the system identification app to best fit the data. The Akaike’s Final Prediction Error (FPE) of the model fits was of the order 10^−2^ for the 5 min sample-and-hold intervals, rising to a maximum of almost 1 for 60 min intervals (see supplementary file S1 Data for full results). An independent input-output model was derived for each microbial population. These were used to tune two independent PI controllers, one controlling each population. We used independent controllers because the PI tuner app is only compatible with single input, single output systems. We considered a range of tuning objectives to assess the merits of tuning to minimise settling time, rise time or overshoot percentage. We found that minimising rise time led to high overshoot errors, while minimising overshoot percentage also led to high errors because the controller would be slow to reach the target. Tuning the controller to minimise settling time worked best for all cases tested and can be seen as a compromise between speed of response and robustness. Hence, for all results presented, the PI controllers were tuned to minimise settling time. All results for the PID tuning, including the gains, FPE, settling times, rise times and overshoot percentages can be found in the supplementary file S1 Data. The Simulink diagram of the system is shown in S10 Fig.

## Supporting information

S1 Fig Episodic Fitted Q-iteration repeats. Population curves of twenty trained agents controlling the chemostat system.
(PDF)

S2 Fig Population curves for different initial conditions and targets. Populations of one of the three replicates for each of the different initial conditions and targets.
(PDF)

S3 Fig Actions for different initial conditions and targets. The actions taken by the agent for the first 600 minutes of one of the three replicates for each of the different initial conditions and targets. The top graph of each panel shows the agent’s actions with respect to the addition of the nutrient that *N*_1_ is dependent on, the bottom graph shows the same for *N*_2_.
(PDF)

S4 Fig Average returns for different initial conditions and targets. Average returns of the three replicates for each of the different initial conditions and targets. Error bars represent one standard deviation.
(PDF)

S5 Fig All population curves from online Fitted Q-iteration running in parallel. Populations curves of five chemostats running in parallel while under online control of a single agent. Here the agent is trained for 1440 minutes (twenty-four hours) and then allowed to control the system for a further 310 minutes to show that the target system behaviour is maintained.
(PDF)

S6 Fig Optimising product output repeats. Population curves of ten trained agents controlling the chemostat system with the goal of optimising product output.
(PDF)

S7 Fig Identifying the minimum timestep above which the chemostat system behaves effectively as a Markov decision process. (A) The error in reward prediction is negligible for time steps above four minutes. Error bars represent one standard deviation. (B) The predicted vs actual reward for one minute and five minute timesteps. Markov decision-based learning is not possible for the short one-minute intervals, but performs well for five-minute intervals.
(PDF)

S8 Fig Overfitting of Fitted Q-iteration for different dataset sizes. The training (blue) and testing (orange) accuracy of a Fitted Q-agent to predict rewards from states and actions was tested after every Fitted Q-iteration. Overfitting is seen for number of transitions less than 200. Error bars represent one standard deviation.
(PDF)

S9 Fig Convergence of Fitted Q-iteration. The scaled error between actual and predicted values as Fitted Q-iterations are completed.
(PDF)

S10 Fig Simulink diagram. The Simulink diagram of the system and PI controllers. (A,B) the setpoints or target population levels, from which the error is calculated (C,D) and used by the PI controllers (E,F) to adjust nutrient levels. The system of ODEs (G) is solved by a continuous time integrator (H).
(PDF)

S1 Data Proportional integral controller data. The performance data resulting from three repeated tunings of a PI controller for all selected sample-and-hold intervals.
(XLSX)
