# Supplementary figures and images for: Deep reinforcement learning for the control of microbial co-cultures in bioreactors
Source: PLoS Comput Biol. 2020 Apr 10;16(4):e1007783. doi: 10.1371/journal.pcbi.1007783 (PMC7176278; doi:10.1371/journal.pcbi.1007783)

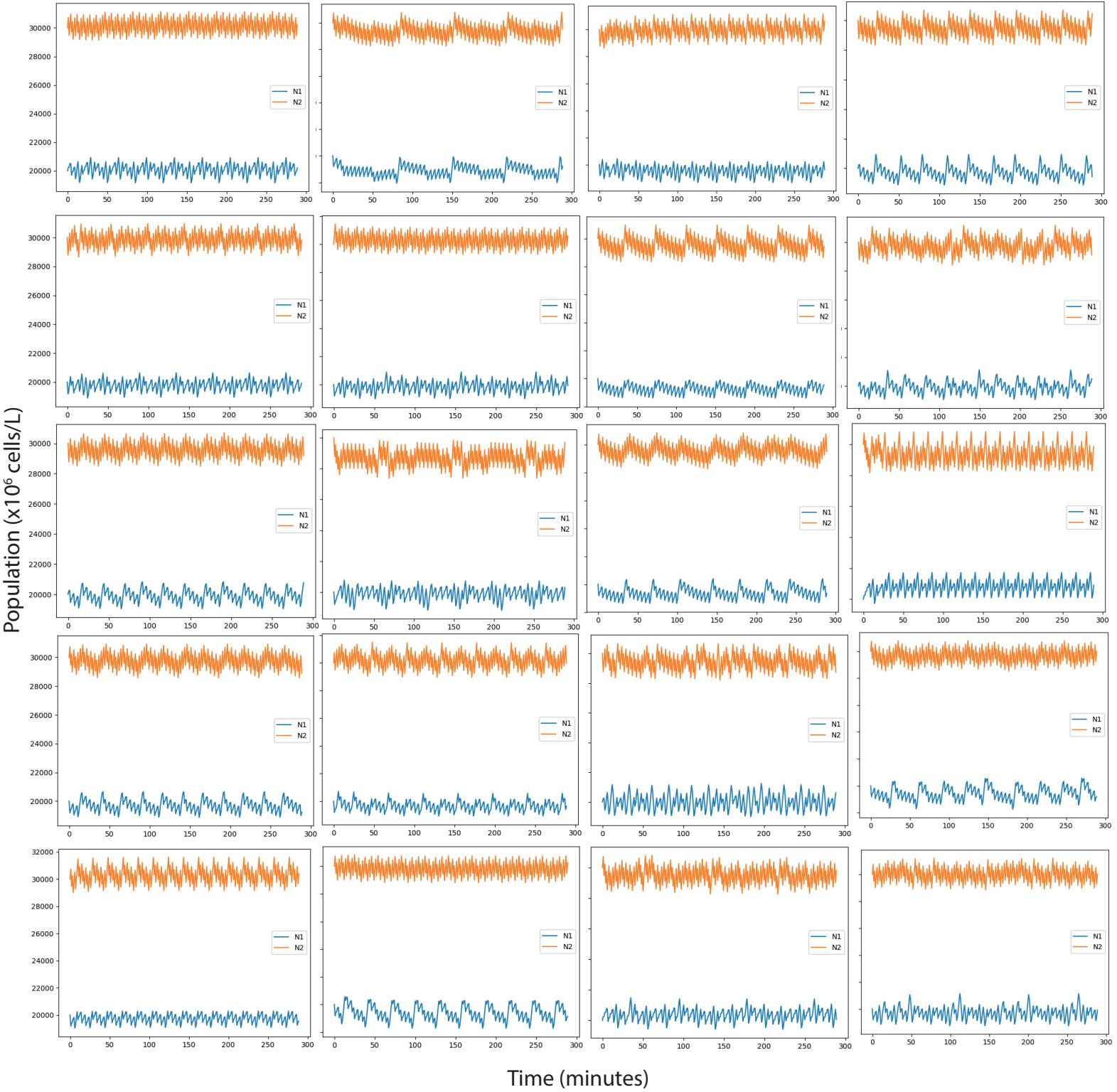

Supplement: S1 Fig — Population curves of twenty trained agents controlling the chemostat system. (PDF) [file pcbi.1007783.s001.pdf]

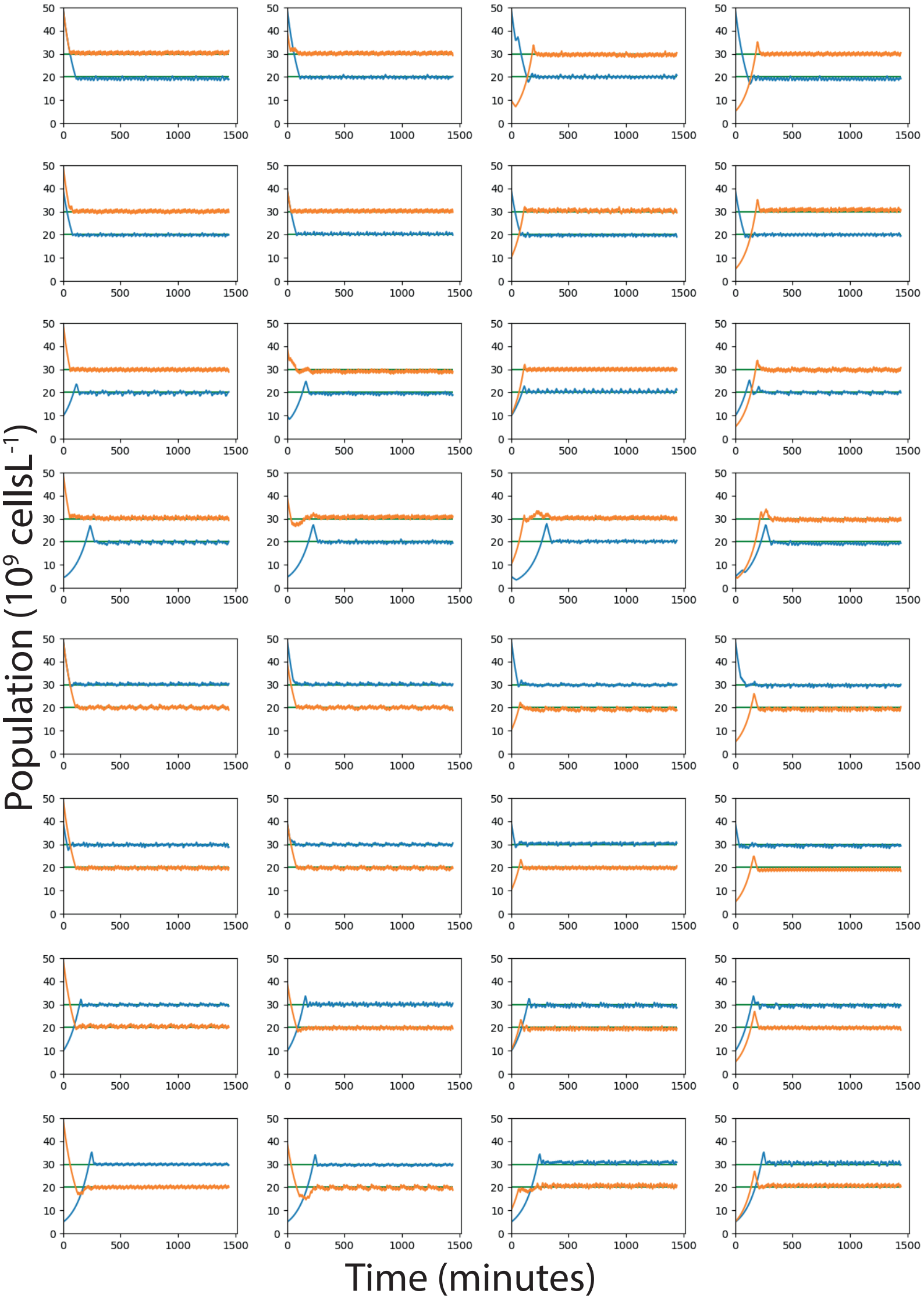

Supplement: S2 Fig — Populations of one of the three replicates for each of the different initial conditions and targets. (PDF) [file pcbi.1007783.s002.pdf]

Action

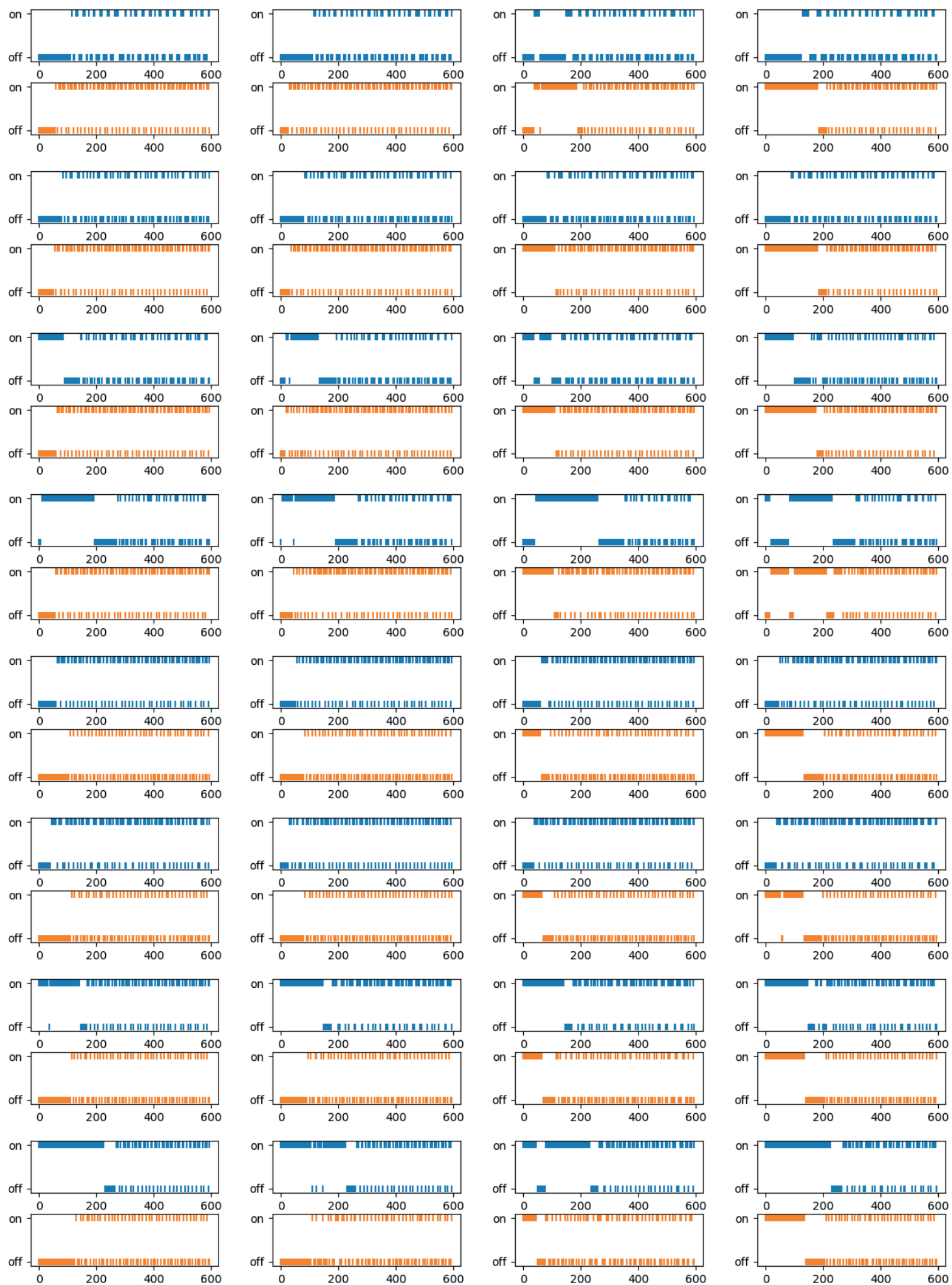

Time (minutes)

Supplement: S3 Fig — The actions taken by the agent for the first 600 minutes of one of the three replicates for each of the different initial conditions and targets. The top graph of each panel shows the agent’s actions with respect to the addition of the nutrient that N1 is dependent on, the bottom graph shows the same for N2. (PDF) [file pcbi.1007783.s003.pdf]

Average return

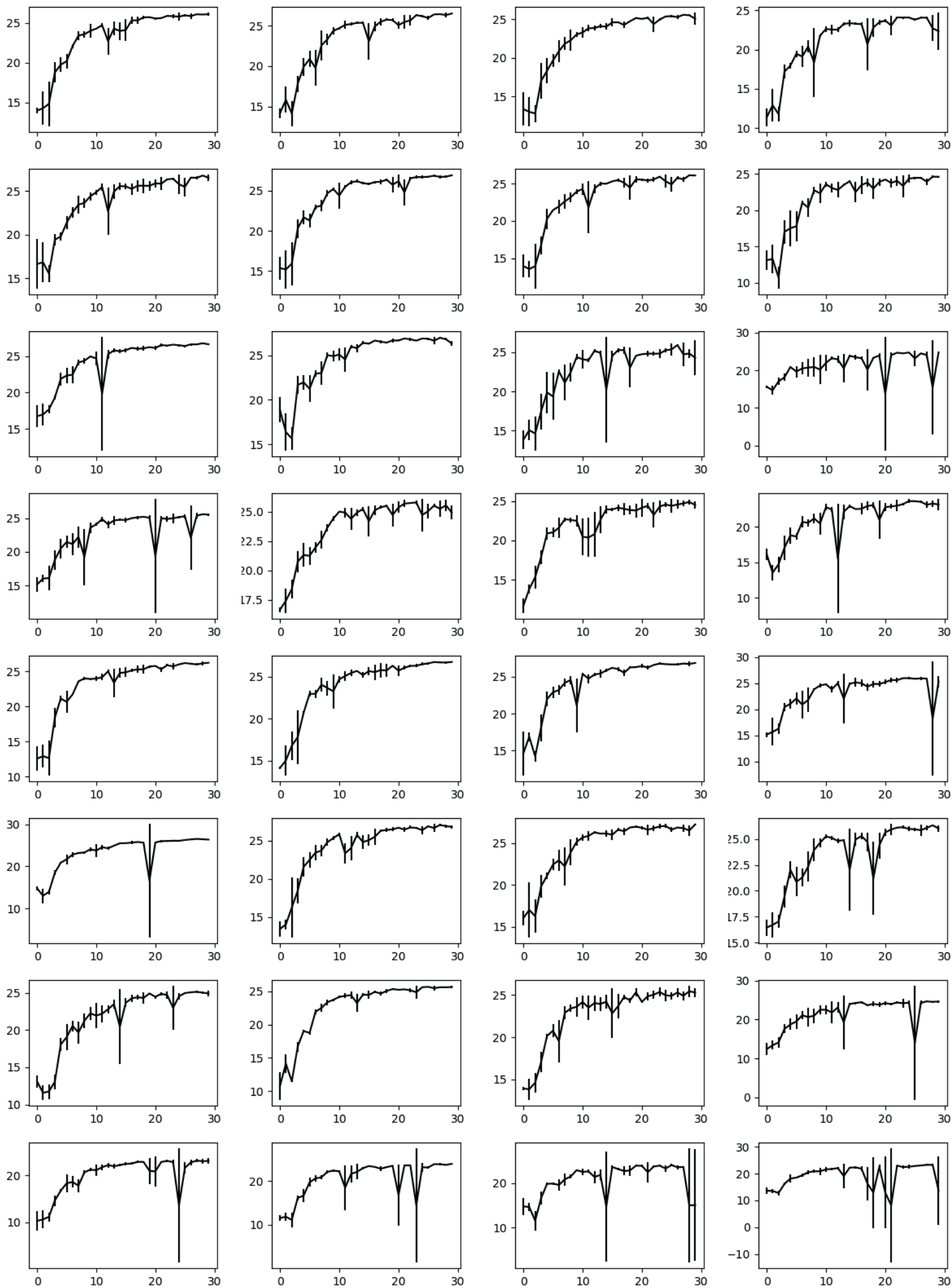

Episode

Supplement: S4 Fig — Average returns of the three replicates for each of the different initial conditions and targets. Error bars represent one standard deviation. (PDF) [file pcbi.1007783.s004.pdf]

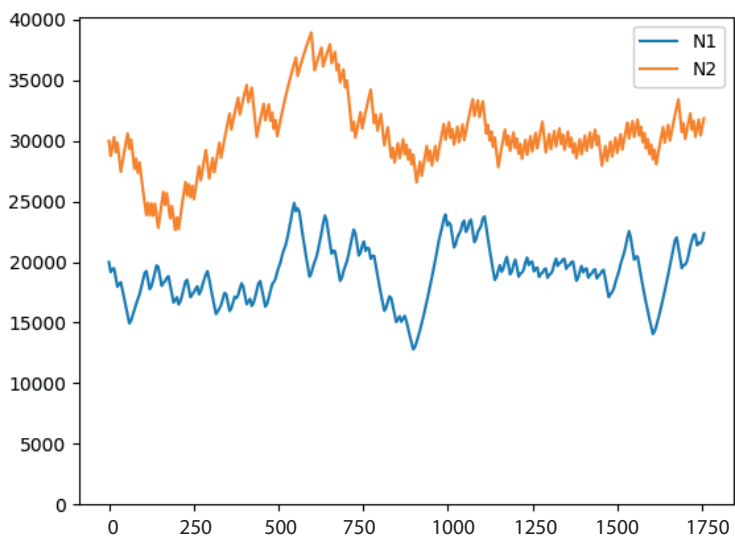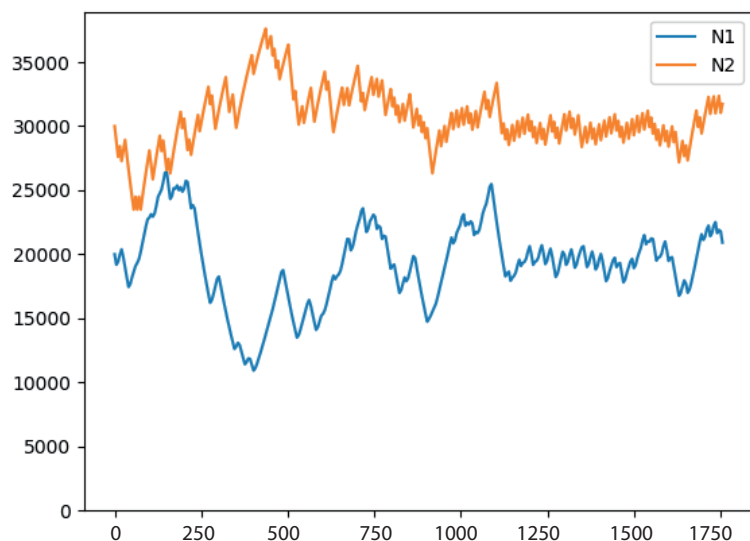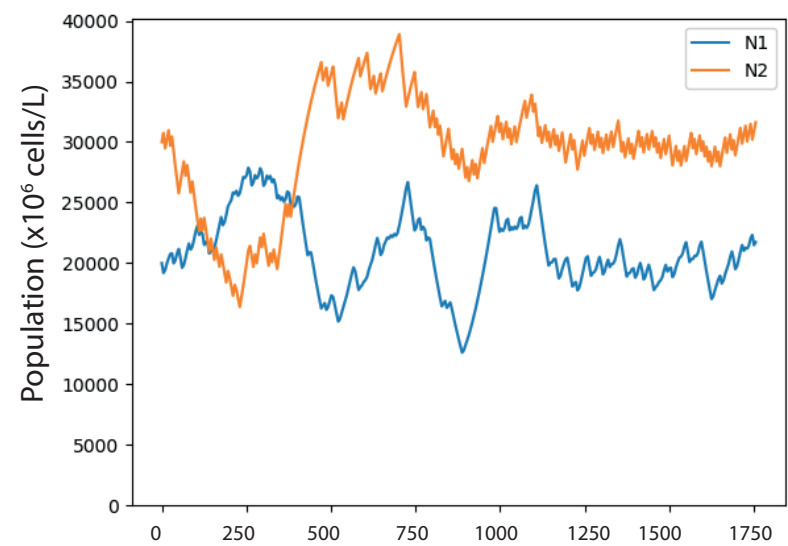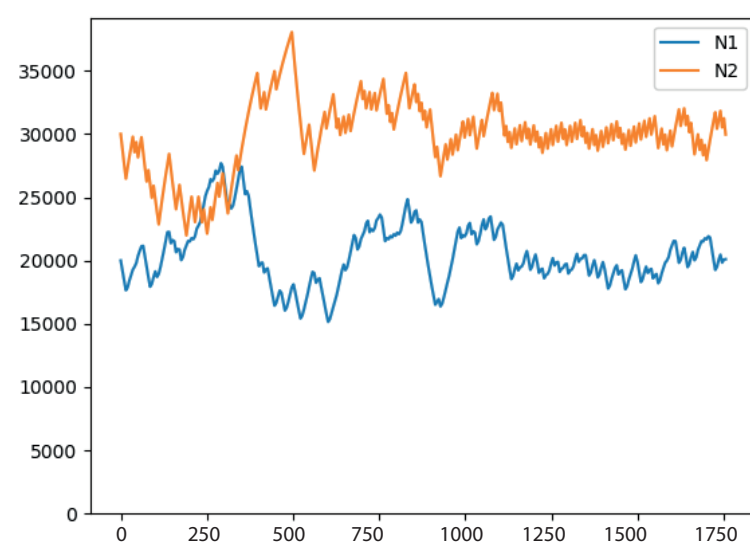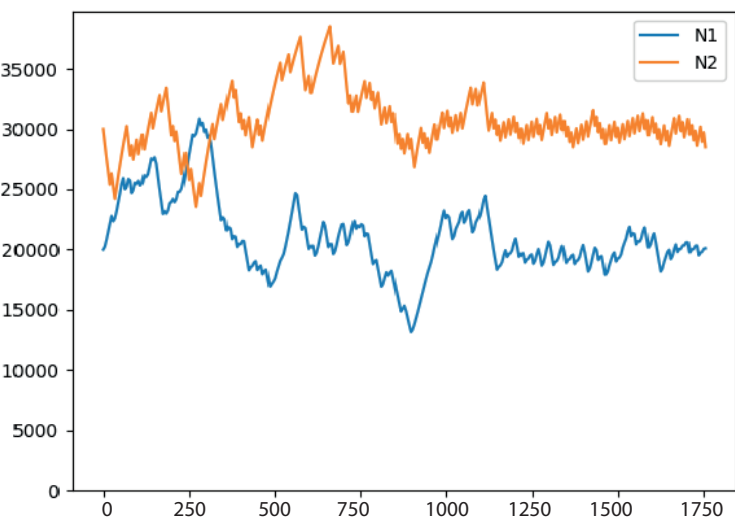

Time (minutes)

Supplement: S5 Fig — Populations curves of five chemostats running in parallel while under online control of a single agent. Here the agent is trained for 1440 minutes (twenty-four hours) and then allowed to control the system for a further 310 minutes to show that the target system behaviour is maintained. (PDF) [file pcbi.1007783.s005.pdf]

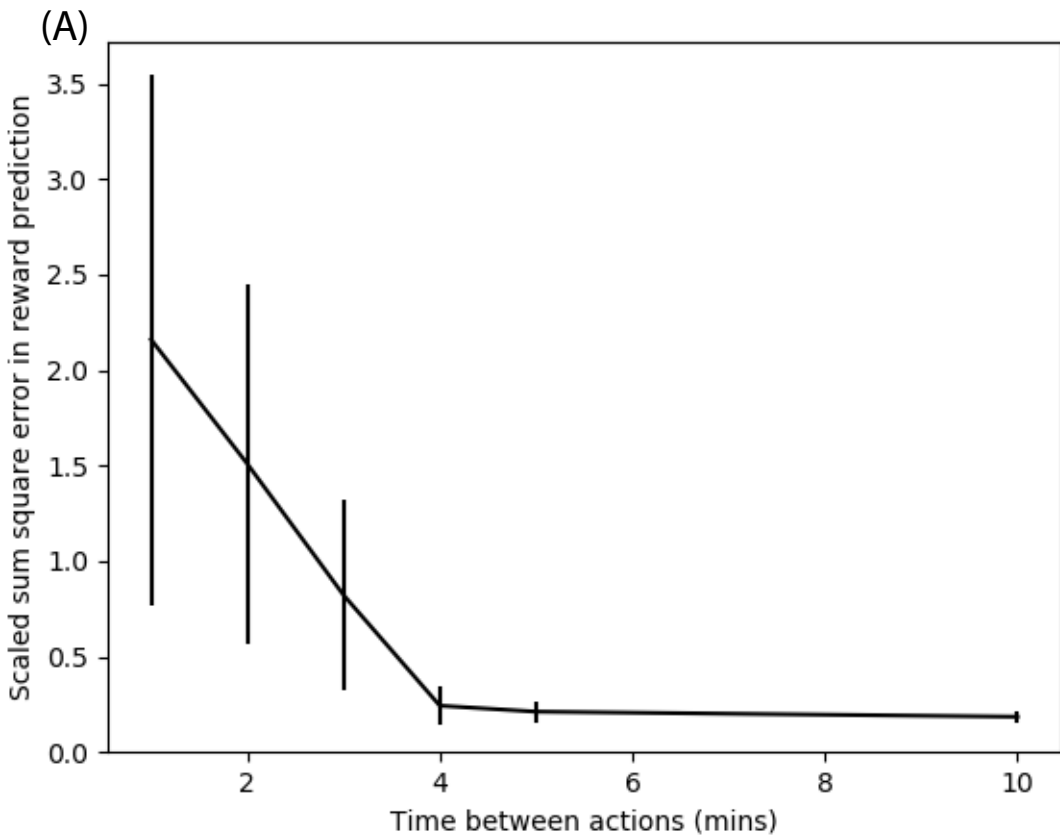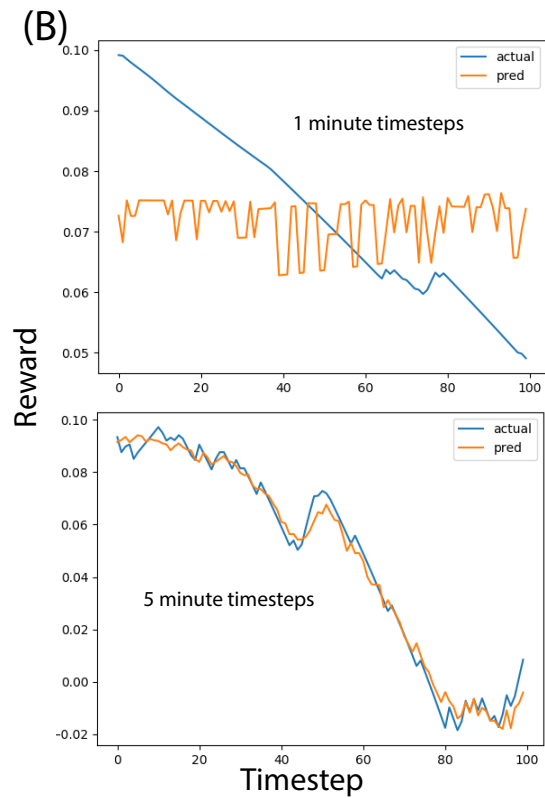

Supplement: S7 Fig — (A) The error in reward prediction is negligible for time steps above four minutes. Error bars represent one standard deviation. (B) The predicted vs actual reward for one minute and five minute timesteps. Markov decision-based learning is not possible for the short one-minute intervals, but performs well for five-minute intervals. (PDF) [file pcbi.1007783.s007.pdf]

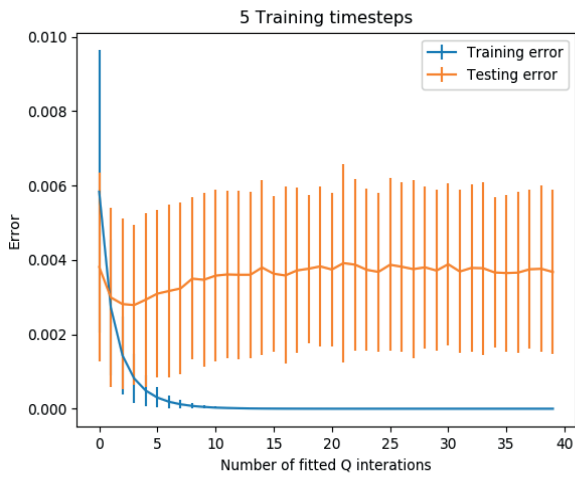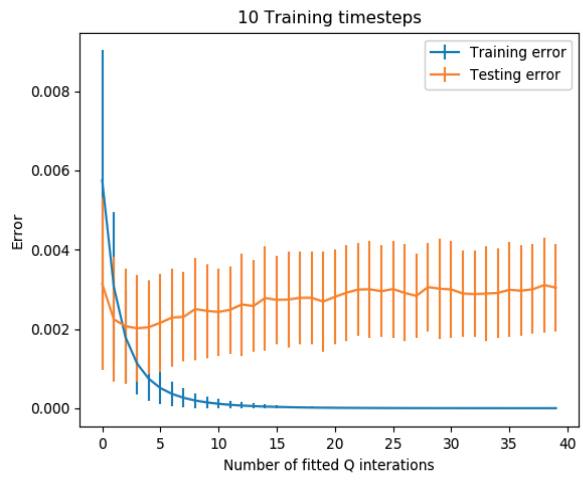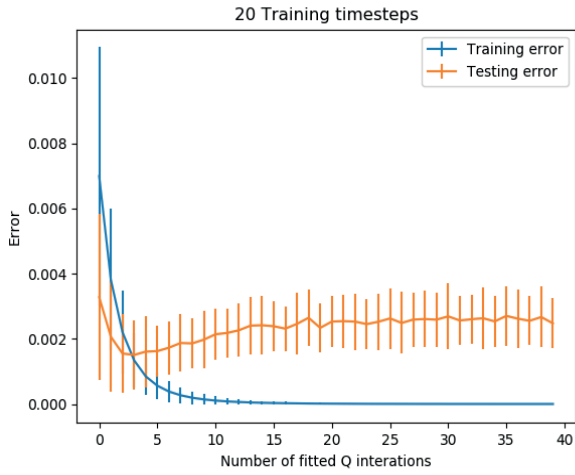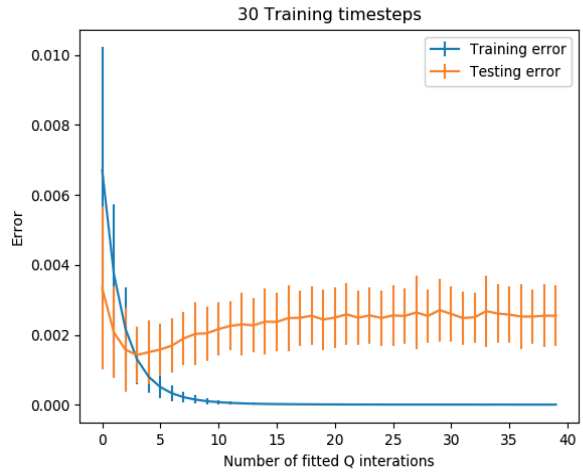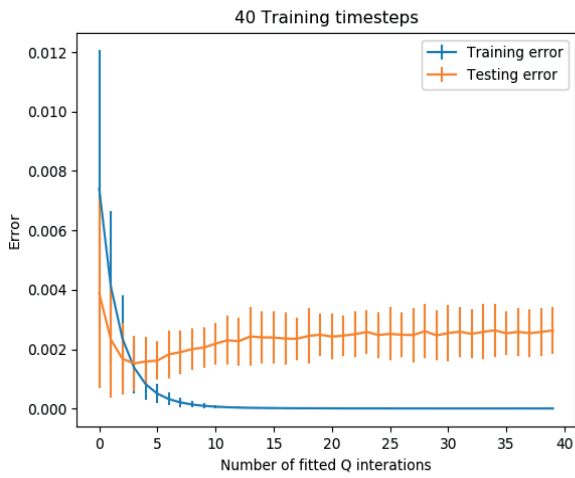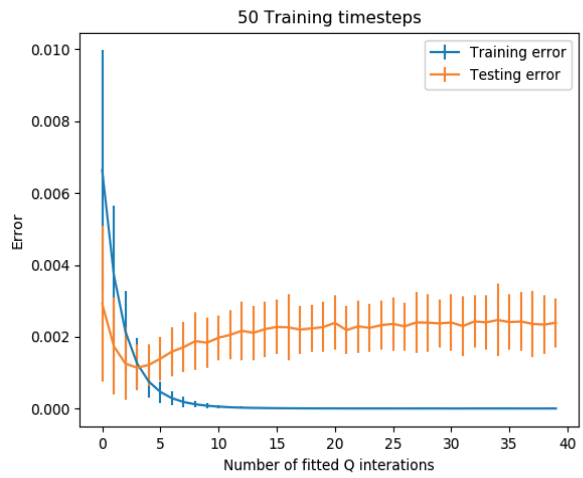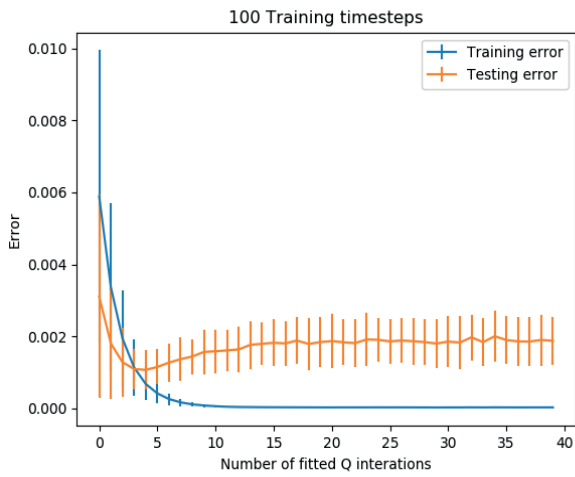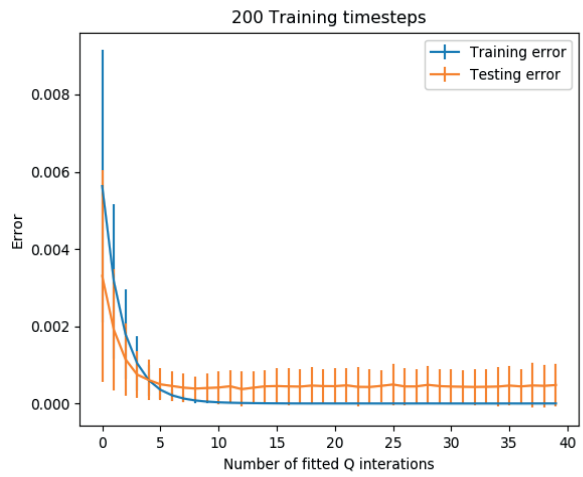

Supplement: S8 Fig — The training (blue) and testing (orange) accuracy of a Fitted Q-agent to predict rewards from states and actions was tested after every Fitted Q-iteration. Overfitting is seen for number of transitions less than 200. Error bars represent one standard deviation. (PDF) [file pcbi.1007783.s008.pdf]

Value Convergence

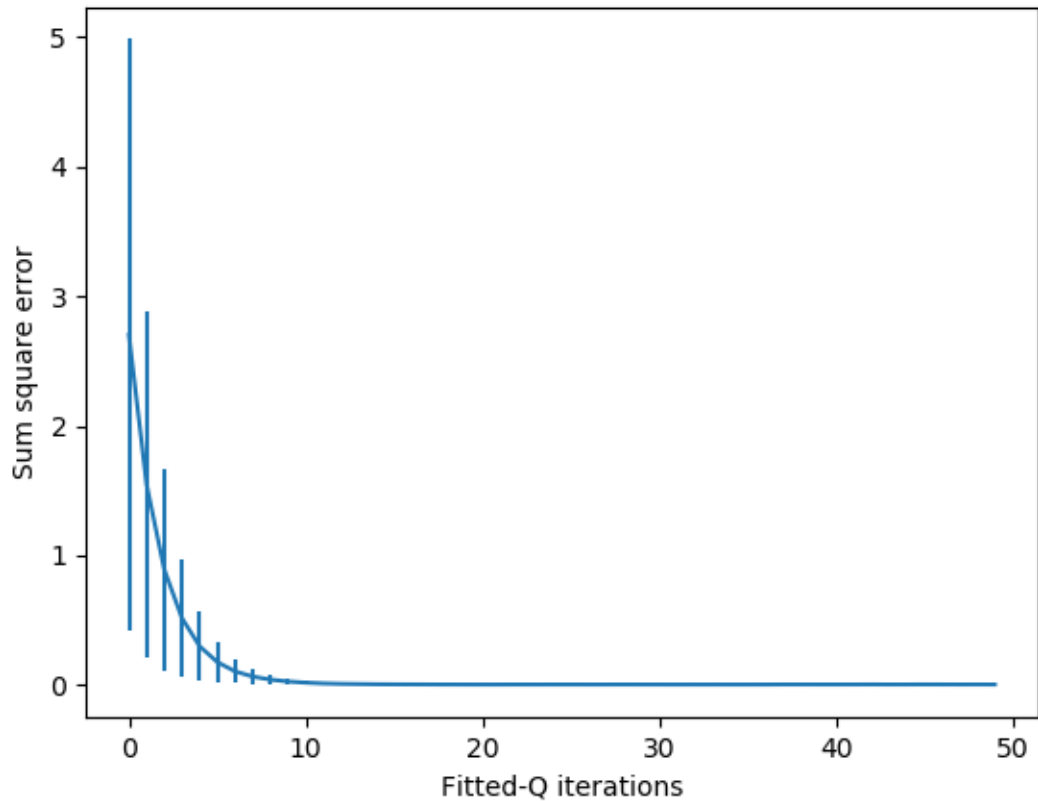

Supplement: S9 Fig — The scaled error between actual and predicted values as Fitted Q-iterations are completed. (PDF) [file pcbi.1007783.s009.pdf]

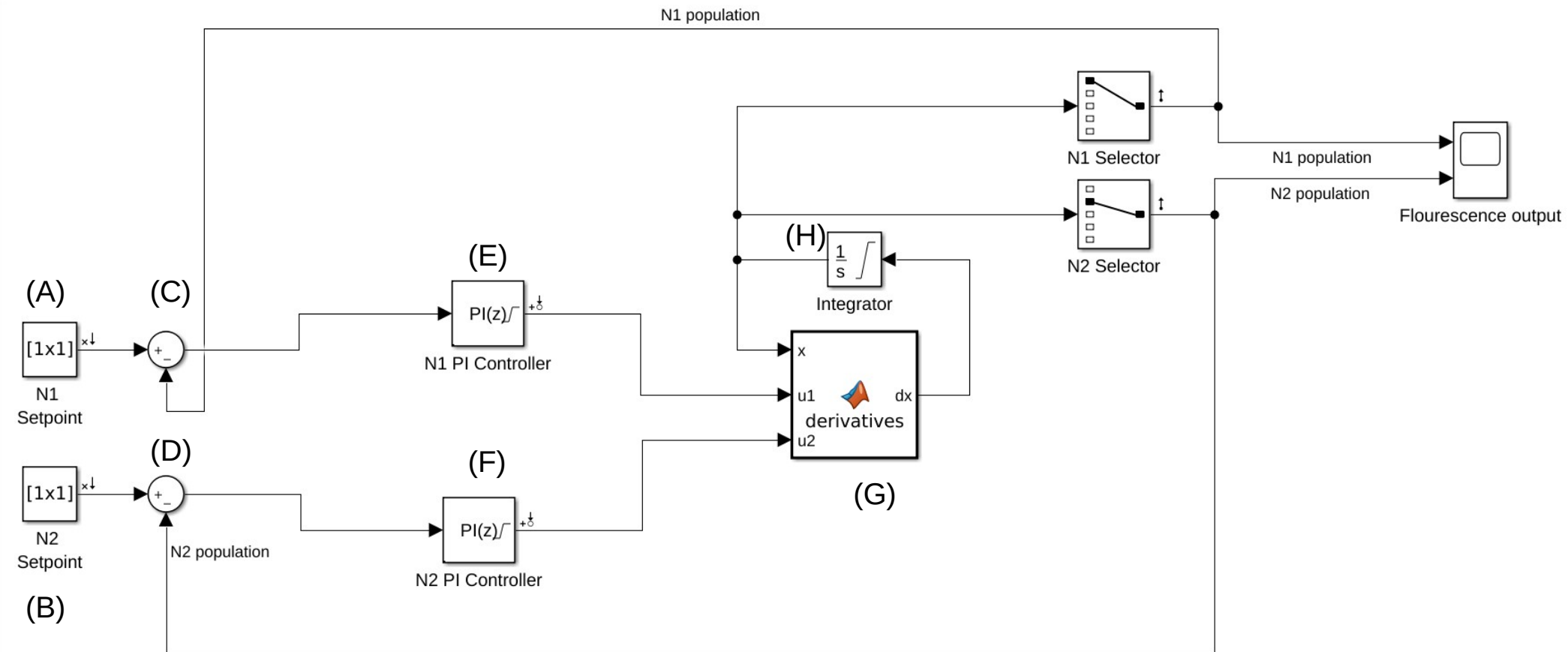

Supplement: S10 Fig — The Simulink diagram of the system and PI controllers. (A,B) the setpoints or target population levels, from which the error is calculated (C,D) and used by the PI controllers (E,F) to adjust nutrient levels. The system of ODEs (G) is solved by a continuous time integrator (H). (PDF) [file pcbi.1007783.s010.pdf]
