# Supplementary material for: Deep reinforcement learning for the control of microbial co-cultures in bioreactors
Source: PLoS Comput Biol. 2020 Apr 10;16(4):e1007783. doi: 10.1371/journal.pcbi.1007783 (PMC7176278; doi:10.1371/journal.pcbi.1007783)
Supplement: S6 Fig — Population curves of ten trained agents controlling the chemostat system with the goal of optimising product output. (PDF) [file pcbi.1007783.s006.pdf]

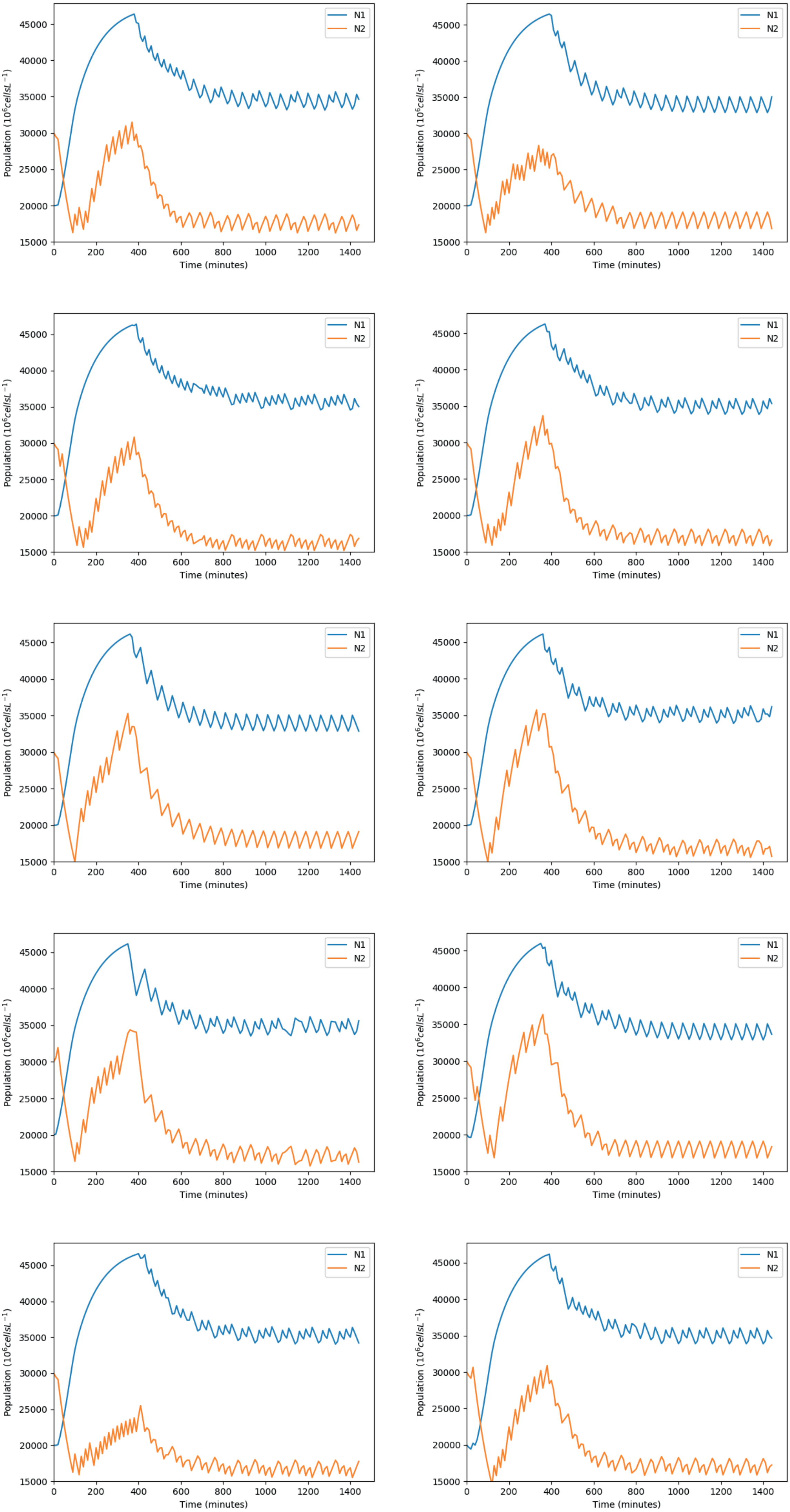
N1
N2
Population ($10^6 cells L^{-1}$)
Time (minutes)
